# Impact of unintended pregnancy on maternal mental health: a causal analysis using follow up data of the Panel Study on Korean Children (PSKC)

**DOI:** 10.1186/s12884-015-0505-4

**Published:** 2015-04-03

**Authors:** Jinwook Bahk, Sung-Cheol Yun, Yu-mi Kim, Young-Ho Khang

**Affiliations:** 1 Institute of Health Policy and Management, Seoul National University College of Medicine, 103 Daehak-ro, Jongno-gu, Seoul 110-799 South Korea; 2 Department of Clinical Epidemiology and Biostatistics, University of Ulsan College of Medicine, Asan Medical Center, 388-1 Poongnap-2dong Songpa-gu, Seoul, 138-736 South Korea; 3 Department of Preventive Medicine, Dong-A University College of Medicine, 26 Daesingongwon-ro, Seo-gu, Busan 602-715 South Korea; 4 Department of Health Policy and Management, Seoul National University College of Medicine, 103 Daehak-ro, Jongno-gu, Seoul 110-799 South Korea

**Keywords:** Inverse probability, Korea, Perinatal depression, Propensity scores, Stress, Unintended pregnancy

## Abstract

**Background:**

Pregnancy intention is important for maternal and child health outcomes. The purpose of this study was to examine the causal relation between pregnancy intention and maternal depression and parenting stress in Korean women who gave birth during 2008.

**Methods:**

This study is a retrospective evaluation of prospectively collected data from the Panel Study on Korean Children from 2008 to 2010. Causal analyses were conducted using propensity score matching and inverse probability of treatment weighted methods. In addition, mediation analyses were performed to test mitigating effects of marital conflict, fathers’ participation in childcare, and mothers’ knowledge of infant development on the relation between unintended pregnancy and adverse maternal mental health.

**Results:**

Results showed that the overall effect of an unintended pregnancy on maternal depression and parenting stress was statistically significant. An unintended pregnancy was associated with 20–22% greater odds of maternal depression, 0.28–0.39 greater depression score, and 0.85–1.16 greater parenting stress score. Relations between pregnancy intention and maternal depression, maternal depression score and parenting stress score were moderately explained by marital conflict and fathers’ participation in childcare.

**Conclusions:**

Unintended pregnancy contributed to increased risks of maternal depression and parenting stress. Efforts to increase fathers’ participation in childcare and decrease marital conflict might be helpful to mitigate adverse impacts of unintended pregnancy on perinatal maternal mental health.

**Electronic supplementary material:**

The online version of this article (doi:10.1186/s12884-015-0505-4) contains supplementary material, which is available to authorized users.

## Background

An unintended pregnancy may be an unwanted pregnancy (did not want to be pregnant at all) or a mistimed pregnancy (pregnancy occurred earlier than wanted), and the term is used interchangeably with unplanned pregnancy [1,2].

Pregnancy intent is an important determinant of both short- and long-term maternal and child health outcomes [3]. Pregnancy intention may affect attitudes and behaviors in parenting and eventually have an impact on child development. Compared to pregnant women with pregnancy intention, pregnant women without pregnancy intention had greater exposure to cigarette smoking, drinking alcohol, taking medications and illicit drugs, and X-ray during pregnancy, and were less likely to take folic acid and attend antenatal care [4-6]. If the pregnancy was intended, babies had a greater likelihood of prolonged breastfeeding and receiving proper vaccinations [7,8]. Pregnancy intention also has long-term influences on child health. Children born after unintended pregnancy showed a cognitive delay at 3-years-old, more behavioral problems at 5- and 7-years-old, and increased problem behaviors and substance use at 14-years-old than their counterparts [9-11]. Unwanted births may also cause poor relations between mother and offspring, even after the child has become an adult [12]. Cleland and colleagues argued that preventing unintended pregnancy by providing family-planning services free of charge is a cost-effective preventive care service [13].

Unintended pregnancy rates vary by country and year, and across sub-populations [14,15]. Singh and colleague estimated that 41% of all pregnancies that occurred worldwide in 2008 were unintended, and the regional prevalence of unintended pregnancies ranged from 30% to 58% [15]. In South Korea (hereafter ‘Korea’), the prevalence of planned pregnancies reported by hospital- and/or community health center-based studies ranges from 51.4% to 74.3% [6,16,17]. A nationwide survey in 2008, the Panel Study on Korean Children (PSKC), reported that 74.3% of pregnancies were intended [16].

An unintended pregnancy is a risk factor for poor maternal mental health including perinatal depression, stress, and lower levels of psychological well-being and life satisfaction [4,18-22]. An unintended pregnancy increases the risk of maternal depression [22-25] and parenting stress [4,26]. However, most prior studies of the consequences of an unintended pregnancy on maternal health were cross-sectional, and few studies were conducted in Asian countries. More importantly, a causal relation between unintended pregnancy and maternal mental health has not been established. Gipson and colleagues argued that the relation between an unintended pregnancy and maternal and child outcomes may be confounded by many factors, including socioeconomic status, and highlighted the need for well-designed studies that provide information on causality [3]. A randomized controlled study on the relation between pregnancy intention and maternal and child outcomes is not possible for ethical reasons. One alternative approach to investigate a causal relation is to perform causal analysis using observational data, such as propensity score matching (PSM) [27].

In this study, we examined the causal relation between pregnancy intention and maternal mental health (maternal depression and parenting stress) by employing PSM and inverse probability of treatment weighted (IPTW) analyses. We used nationally representative, prospective birth cohort data collected in Korea from 2008 to 2010, and we used repeated measures of maternal mental health obtained over the study period. We hypothesized that unintended pregnancy would have a strong adverse effect on maternal mental health in the early stages of postpartum but that the strength of this effect would diminish with time. This hypothesis was partly based on our assumption that unintended pregnancy may cause short-term adverse mental health problems because of its nature, i.e., unintended pregnancy being an unexpected incident and burden in the mother’s life, but that growing mother-infant attachment and intimacy after birth would minimize this initial effect. This hypothesis was also partly based on literature showing an effect of pregnancy intention on antenatal and postpartum maternal mental health [4,18,21,23] but no effect of pregnancy intention on maternal mental health in later stages of parenting [28-30]. We also hypothesized that marital conflict, fathers’ participation in childcare, and mothers’ knowledge of infant development would mediate the relation between pregnancy intention and maternal mental health. This second hypothesis is supported by prior studies on the effects of such mediators on maternal mental health [31,32].

## Methods

### Data

We used publicly available data from the 2008–2010 PSKC conducted by the Korea Institute of Child Care and Education. The PSKC is a longitudinal survey on a representative national sample of children born between April and July 2008 and their parents. Participants were recruited from 30 sampled hospitals across the country. The first wave of PSKC was conducted in 2008, and follow-up surveys have been performed annually. The initial interview was face-to-face and was conducted at the time of childbirth in the hospital. A subsequent telephone survey (the second survey of the first wave of PSKC) was conducted at 1 month postpartum. The third survey was initiated and conducted at the participant’s home at 4 months postpartum. The mean time of the third survey was 5.6 months after birth (standard deviation (SD) = 1.2 months). These three surveys (at birth, 1 month postpartum, and 4 months postpartum) were conducted in 2008 and comprise the first wave of PSKC. The second and third waves of PSKC were face-to-face interviews conducted in the participant’s home at 1 year postpartum (mean = 14.1 months; SD = 1.1 months) and 2 years postpartum (mean = 25.8 months; SD = 1.4 months). The number of responding families was 2,078 in the first wave, 1,904 in the second wave, and 1,802 in the third wave. This study was approved by the Asan Medical Center Institutional Review Board. Written informed consent was obtained from each participant at the time of recruitment by the Korea Institute of Child Care and Education.

### Pregnancy intention

Pregnancy intention was determined in the initial interview (i.e., the first survey of the first wave) by asking the mother “Did you and your husband (or partner) plan the pregnancy or want to have the baby?” Response categories were: 1) only I as the mother of the baby planned or wanted the pregnancy, 2) only my husband (or partner) as the father of the baby planned or wanted the pregnancy, 3) both my husband (or partner) and I planned or wanted the pregnancy, and 4) neither my husband (or partner) nor I planned or wanted the pregnancy. In this study, we focused on the mother’s intention, as we viewed this as more directly related to maternal mental health than the father’s intention. Therefore, we grouped responses 1) and 3) as intended pregnancy, and responses 2) and 4) as unintended pregnancy.

### Depression

Maternal depression was assessed using the Kessler 6-Item Psychological Distress Scales (K6). The K6 is a short screening instrument for mental illness in the general population. In the PSKC, the response to each of the six items was scored on an ordinal scale from 1 to 5, and the total score ranged from 6 to 30. An additional file shows full questionnaires [see Additional file 1]. The reliability of the K6 is high (Cronbach alpha = 0.89) [33]. Subjects scoring ≥14 were classified as depressed [34-36]. Maternal depression was measured five times during the study period: at the time of childbirth, and at 1 month, 4 months, 1 year and 2 years postpartum). The survey conducted at the time of childbirth asked mothers about their feelings during the 1 month prior to giving birth (antenatal depression), and the surveys conducted at 1 month, 4 months, 1 year and 2 years postpartum asked mothers about their feelings during the preceding 30 days. The same six items and response categories were used throughout the study period.

### Parenting stress

The mothers’ parenting stress was measured with ten questions on perceived difficulties and distress in taking care of the baby and performing parental roles (see questionnaires in Additional file 1). These ten questions originated from a Korean study [37] and were developed based on the Parenting Stress Index [38-40], Parenting Daily Hassles [41], and Maternal Guilt Scale [42]. The reliability of the questions is high (Cronbach alpha = 0.88) [37]. The ten items focus on negative psychological states that arise from parenting demands. Each of ten items is scored on a five-point Likert scale ranging from 1 to 5. The total score ranges from 10 to 50, with higher scores indicating greater maternal parenting stress [37]. Parenting stress was measured at three time points during the study period: 4 months, 1 year, and 2 years postpartum. The same ten items and response categories were used throughout the study period.

### Mediators

We selected three variables as potential mediators of the relation between pregnancy intention and maternal mental health: marital conflict, father’s participation in childcare, and mother’s knowledge on infant development. Mediators evaluated by the mother at 4 months postpartum were used in the analysis.

#### Marital conflict

We hypothesized that women with an unintended pregnancy may experience marital conflict with their husband that was related to unexpected pregnancy, and therefore were more likely to experience marital conflict than women with an intended pregnancy. Marital conflict was evaluated using the Korean version of the marital conflict index (see questionnaires in Additional file 1), which consists of eight items regarding conflictual situations including escalation, invalidation, negative interpretations, withdrawal and avoidance between couples. This index was derived from Markman et al.’s relation dynamics scale [43] translated by Chung [44]. The reliability of the index is high (Cronbach alpha = 0.96) [44]. Each item is scored on a five-point Likert scale ranging from 1 to 5. The total score ranges from 8 to 40, with higher scores indicating a greater level of marital conflict.

#### Fathers’ participation in childcare

We hypothesized that women with an intended pregnancy would report greater participation of the father in childcare than women with an unintended pregnancy. The mother was asked about the cooperation of the child’s father in childcare using four items from the husband’s family role performance questionnaire developed by Hong [45] that were extracted by a previous study [46]. Each item is scored on a five-point Likert scale ranging from 1 to 5. The total score ranges from 4 to 20, with higher scores indicating more involvement of the child’s father in childcare. The reliability of the questions is high (Cronbach alpha = 0.86) [33].

#### Knowledge of infant development

We hypothesized that women with an intended pregnancy would be more likely to gather information on infant development than women with an unintended pregnancy, and thus would be more knowledgeable on infant development than women with an unintended pregnancy. Knowledge of infant development was assessed using the Knowledge of Infant Development Inventory, which was developed to measure the mothers’ knowledge of childrearing and child development [47]. The original Knowledge of Infant Development Inventory consists of four sub-sections: norms and milestones, parenting strategies, principles of development, and health and safety. The PSKC used 13 items from the principles of development sub-section. Mothers responded to each item by yes or no, or reporting they were not sure. Total score was computed by the sum of the number of items correctly answered.

### Socio-demographic characteristics

Socio-demographic characteristics evaluated at 4 months postpartum (baseline) were used in the analysis. Maternal and paternal education were categorized as high school or under, junior college, or university or over. Maternal occupation was categorized as non-manual, manual, or housewife/other, and paternal occupation was categorized as non-manual or manual. Household income was adjusted for family size and then divided into quintiles. Maternal and paternal cigarette smoking and alcohol drinking behaviors (yes or no) were also assessed at 4 months postpartum. The sex and birth order (first born vs. later born) of the infant were also recorded.

### Statistical analysis

Categorical variables are presented as frequencies and percentages and were compared across women with intended pregnancies and women with unintended pregnancies using the chi-square test or Fisher’s exact test. Continuous variables are expressed as mean ± standard deviation (SD) and were compared across women with intended pregnancies and women with unintended pregnancies using Student’s unpaired t-test. Logistic and linear regression analyses were conducted for each time point to evaluate the effect of pregnancy intention on each outcome, and generalized linear mixed models were used to examine time trends in the effects of pregnancy intention.

PSM and IPTW were used for causal analyses. Propensity score analysis and regression based approaches can be used to estimate treatment effects in observational data. Several advantages of propensity score analysis over regression based approaches exist. For example, propensity score analysis can be used to reduce or eliminate the effects of confounding when using observational data to estimate treatment effects [27]. To reduce the effect of selection bias and potential confounding, differences in baseline characteristics (maternal age, paternal age, infant’s sex, infant’s birth order, maternal education, paternal education, maternal occupation, paternal occupation, household income, maternal smoking, paternal smoking, maternal alcohol consumption, paternal alcohol consumption) were adjusted using weighted generalized linear mixed models with inverse probability of treatment weighting [48]. With this technique, weights for women with unintended pregnancy were the inverse of the propensity score and weights for women with intended pregnancy were the inverse of 1 - propensity score. The propensity score is the probability, given baseline variables, that any participant in either group would be selected for unintended pregnancy. The propensity scores were estimated without regard to outcomes by multiple logistic regression analysis. A full non-parsimonious model was developed that included all variables shown in Table 1. Model discrimination was assessed with C statistics (C = 0.618) and model calibration was assessed with Hosmer-Lemeshow statistics *(p* = 0.3340). The results of IPTW were verified by PSM. The propensity score-matched pairs (one-to one matching) were created by matching women with unintended and intended pregnancies on the logit of the propensity score using calipers of width equal to 0.2 of the SD of the logit of the propensity score. After propensity score matching, we examined the similarity of women with unintended and intended pregnancies in the propensity score-matched sample by calculating standardized differences for each of the baseline variables listed in Table 1. All of the standardized differences for each of the baseline variables were less than 0.06 (6%) (See Additional file 1: Table S1). Mediation analyses were conducted to test the hypotheses that marital conflict, fathers’ participation in childcare, and mothers’ knowledge of infant development mediated the relation between pregnancy intention and outcomes. The role of mediators in the relation between pregnancy intention and the presence of maternal depression was evaluated using the percentage excess odds explained by inclusion of the mediators in the model, which was calculated as (OR_baseline model_ – OR_baseline model + mediators_)/(OR_baseline model_ – 1) [49]. Similarly, the role of mediators in the relation between pregnancy intention and maternal depression score and parenting stress score was evaluated using the percentage excess beta explained by inclusion of the mediators in the model, which was calculated as (Beta_baseline model_ – Beta_baseline model + mediators_)/(Beta _baseline model_). This excess odds or beta explained the degree to which a mediator explains the relation between pregnancy intention and maternal mental health. All statistical analyses were performed with SAS version 9.1 (SAS Institute, Cary, NC). A two-tailed value of *p* < 0.05 was considered statistically significant.

**Table 1:** **Baseline characteristics of study subjects according to pregnancy intention**

	Total	Pregnancy intention		
		Unintended	Intended	
	N = 2076	N = 525 (25.3%)	N = 1551 (74.7%)	P value
Maternal age (years)	31.3 ± 3.7	30.0 ± 4.2	31.5 ± 3.5	0.0228
Paternal age (years)	33.9 ± 4.0	33.8 ± 4.6	33.9 ± 3.8	0.5571
Infant’s sex				
Boy	1056 (50.9)	262 (49.9)	794 (51.2)	0.6099
Girl	1020 (49.1)	263 (50.1)	757 (48.8)	
Infant’s birth order				
First born	984 (47.5)	200 (38.1)	784 (50.7)	<0.0001
Later born	1087 (52.5)	325 (61.9)	762 (49.3)	
Maternal education				
University or over	829 (40.2)	193 (37.1)	635 (41.2)	0.0268^§^
Junior college	589 (28.6)	144 (27.7)	445 (28.9)	
High school or under	643 (31.2)	183 (35.2)	460 (29.9)	
Paternal education				
University or over	1003 (50.4)	234 (46.4)	769 (51.7)	0.0356^§^
Junior college	420 (21.1)	111 (22.0)	309 (20.8)	
High school or under	568 (28.5)	159 (31.6)	409 (27.5)	
Maternal occupation				
Non-manual	516 (25.2)	127 (24.4)	389 (25.5)	0.5975
Manual	113 (5.5)	33 (6.4)	80 (5.3)	
Housewife/others	1415 (69.2)	360 (69.2)	1055 (69.2)	
Paternal occupation				
Non-manual	1261 (65.6)	299 (61.8)	962 (66.8)	0.0421
Manual	662 (34.4)	185 (38.2)	477 (33.2)	
Household income quintiles				
I (highest)	411 (19.9)	89 (17.1)	322 (20.9)	0.0002^§^
II	445 (21.6)	95 (18.2)	350 (22.7)	
III	394 (19.1)	100 (19.2)	294 (19.1)	
IV	377 (18.3)	104 (19.9)	273 (17.7)	
V (lowest)	436 (21.1)	134 (25.7)	302 (19.6)	
Maternal smoking				
No	2059 (99.2)	519 (98.9)	1540 (99.3)	0.399*
Yes	17 (0.8)	6 (1.1)	11 (0.7)	
Paternal smoking				
No	1174 (56.6)	263 (50.1)	911 (58.7)	0.0006
Yes	902 (43.5)	262 (49.9)	640 (41.3)	
Maternal alcohol consumption				
No	1359 (73.0)	347 (72.4)	1012 (73.2)	0.7388
Yes	502 (27.0)	132 (27.6)	370 (26.8)	
Paternal alcohol consumption				
No	367 (21.4)	100 (22.5)	267 (21.0)	0.4931
Yes	1350 (78.6)	344 (77.5)	1006 (79.0)	

*Fisher’s exact test. ^§^Mantel-Haenszel Chi-square test. Data are mean ± standard deviation or n (%).

## Results

Table 1 shows the neonatal and socio-demographic characteristics of study subjects according to pregnancy intention. Of 2076 pregnancies, 525 (25.3%) were unintended. The prevalence of unintended pregnancy was significantly higher among later-born infants, fathers with a manual job, lower household income, and fathers who were smokers than their counterparts (Table 1).

**Figure 1:**
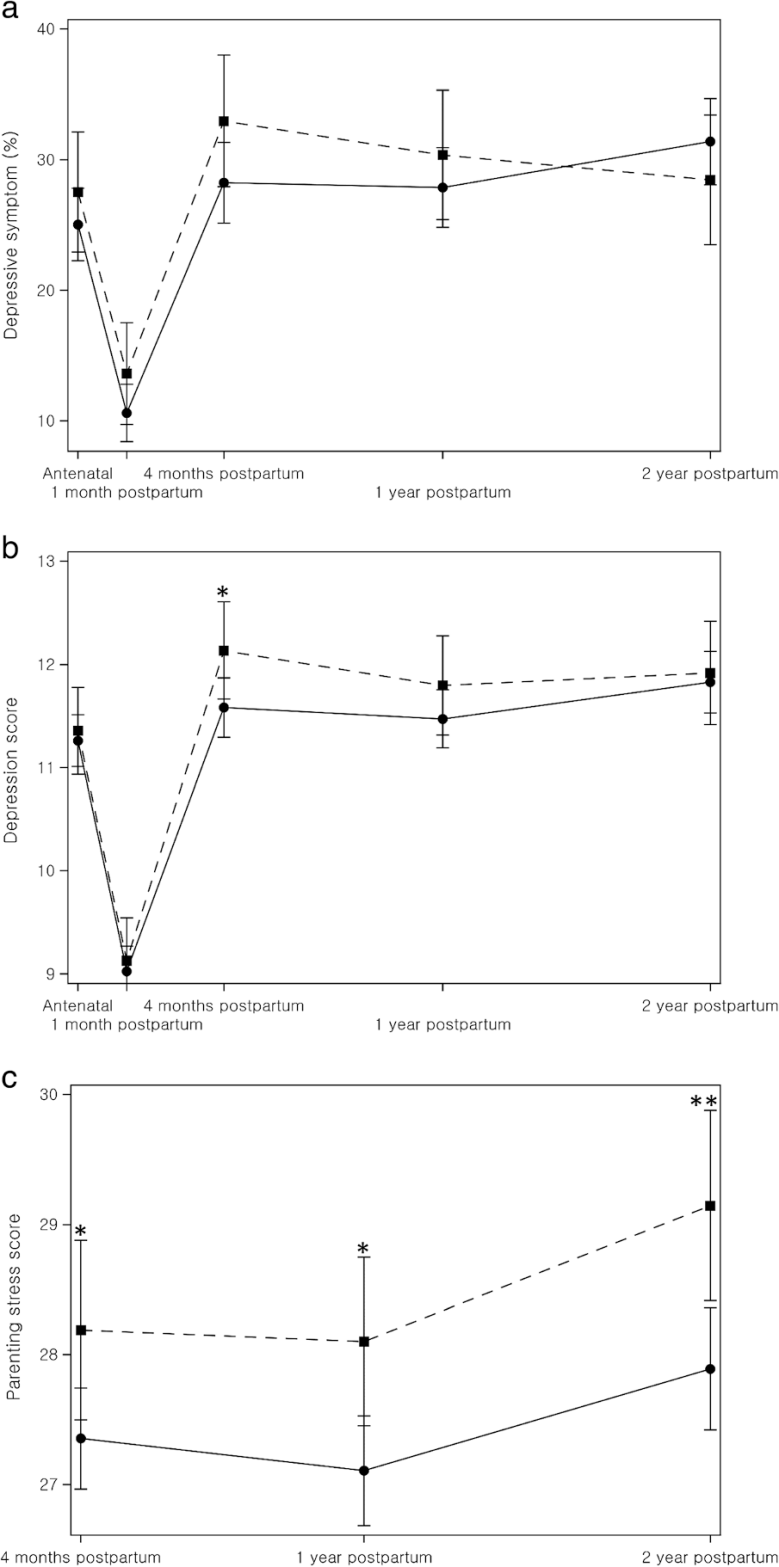
**Distribution of maternal mental health status according to pregnancy intention.** ● Intended ■ Unintended. Note. **(a)** the prevalence of maternal depressive symptoms, **(b)** the mean maternal depression score, and **(c)** the mean maternal parenting stress score according to pregnancy intention. Data are adjusted for maternal age, birth order, maternal education, paternal occupation and household income. Error bars indicate 95% confidence interval. *P < 0.05, **P < 0.01.

Figure 1 shows the prevalence of maternal depression (Figure 1a), the maternal depression score (Figure 1b), and the parenting stress score (Figure 1c) according to pregnancy intention over the study period. In both groups, the prevalence of maternal depression and the average maternal depression score decreased shortly after delivery (at 1 month postpartum) and then returned to the antenatal level at 3 months postpartum (Figure 1a and b). At the first four time points, women with an unintended pregnancy had a higher prevalence of depression than women with an intended pregnancy, but this difference had disappeared by the final time point (2 years postpartum; Figure 1a). However, the difference of prevalence of depression between two groups was not statistically significant over the study period. At 4 months postpartum, the depression score was higher in women with an unintended pregnancy than in women with an intended pregnancy (p = 0.0397; Figure 1b). At 4 months, 1 year and 2 years postpartum, the parenting stress score was higher in women with an unintended pregnancy than in women with an intended pregnancy (p = 0.0351, p = 0.0113, p = 0.0043), and in both groups the parenting stress score increased at 2 years postpartum (Figure 1c).

Table 2 shows the scores for marital conflict, fathers’ participation in childcare, and mothers’ knowledge of infant development in women with intended and unintended pregnancy. Women with an unintended pregnancy reported significantly greater levels of marital conflict than women with an intended pregnancy and lower participation of the child’s father in childcare. Women with an unintended pregnancy showed a tendency for lower knowledge of infant development than their counterparts, but the difference was not statistically significant.

**Table 2:** **Adjusted mean scores of potential mediating variables according to pregnancy intention**

	Unintended pregnancy	Intended pregnancy	P value
Marital conflict	16.83 (16.25-17.42)	15.78 (15.42-16.13)	0.0019
Fathers’ participation in child care	13.85 (13.55-14.16)	14.48 (14.30-14.66)	0.0004
Knowledge of infant development	8.59 (8.40-8.78)	8.74 (8.62-8.85)	0.1904

Data are mean (95% confidence interval). All variables were evaluated by mothers. Data are adjusted for maternal age, birth order, maternal education, paternal occupation and household income.

Tables 3 and 4 show the results of PSM and IPTW analyses. The odds ratios for maternal depression varied over the survey period and, in PSM analysis, were statistically significant at the early time points (antenatal and the first month postpartum) but not at the 1 and 2 year postpartum time points. However, the interaction between pregnancy intention and time point was not statistically significant in the PSM nor the IPTW analyses (p = 0.1819, p = 0.2402). The overall effect of an unintended pregnancy on the presence of maternal depression was statistically significant in both analyses, with an unintended pregnancy associated with 22% or 20% greater odds of maternal depression in PSM and IPTW analyses, respectively (Table 3). We conducted additional analysis to calculate the difference in maternal depression score according to pregnancy intention. Additional file 1: Table S3) shows the results of PSM and IPTW analyses for maternal depression score, and the findings were similar as those for the presence of maternal depression. Maternal depression score was higher in women with unintended pregnancy than in women with intended pregnancy at the early time points (antenatal depression score in PSM analysis, and 1 month and 4 months postpartum depression scores in the IPTW analysis). The interaction between pregnancy intention and time point was not statistically significant. The overall effect of an unintended pregnancy on maternal depression score was statistically significant (Additional file 1: Table S3).

**Table 3:** **Odds ratio of maternal depressive symptoms according to pregnancy intention**

	PSM			IPTW		
	OR	95% CI	P value	OR	95% CI	P value
Time of inquiry						
Antenatal*	1.32	1.01–1.72	0.0394	1.17	0.93–1.47	0.1781
1 month postpartum	1.59	1.06–2.40	0.0265	1.48	1.05–2.07	0.0252
4 months postpartum	1.30	0.98–1.71	0.0685	1.36	1.08–1.72	0.0101
1 year postpartum	1.19	0.90–1.56	0.2203	1.15	0.91–1.46	0.2394
2 years postpartum	0.95	0.71–1.28	0.7390	1.01	0.79–1.30	0.9215
Pregnancy intention x time of inquiry^§^			0.1819			0.2402
Overall pregnancy intention	1.22	1.02–1.46	0.029	1.20	1.03–1.40	0.0212

*Antenatal depression was measured immediately after childbirth. ^§^The interaction between pregnancy intention and time of inquiry.

PSM: propensity score matching; IPTW: inverse probability of treatment weighted; OR: odds ratio; CI: confidence interval.

**Table 4:** **Difference in parenting stress scores according to pregnancy intention**

	PSM			IPTW		
	β (SE)	95% CI	P value	β (SE)	95% CI	P value
**Parenting stress score**						
Time of inquiry						
4 months postpartum	0.78 (0.40)	0.00–1.57	0.0502	1.15 (0.35)	0.47–1.82	0.0009
1 year postpartum	1.09 (0.41)	0.29–1.88	0.0076	1.24 (0.35)	0.56–1.92	0.0004
2 years postpartum	0.88 (0.43)	0.05–1.71	0.0387	1.35 (0.38)	0.61–2.08	0.0003
Pregnancy intention x time of inquiry^§^			0.8987			0.9955
Overall pregnancy intention	0.85 (0.21)	0.44–1.27	<0.0001	1.16 (0.30)	0.58–1.74	<0.0001

^§^The interaction between pregnancy intention and time of inquiry. PSM: propensity score matching; IPTW: inverse probability of treatment weighted; CI: confidence interval.

Table 4 shows the results of PSM and IPTW analyses for parenting stress scores. In contrast to the findings for the presence of maternal depression and maternal depression score, the differences in parenting stress score between groups was statistically significant throughout the study periods, and the interaction between pregnancy intention and time point was not statistically significant. There was a statistically significant overall effect of pregnancy intention on parenting stress scores in both PSM and IPTW analyses (Table 4).

Table 5 presents the results of analyses on the role of marital conflict, fathers’ participation in childcare, and mothers’ knowledge of infant development on the relation between pregnancy intention and maternal mental health. In both PSM and IPTW analysis, odds ratios decreased with adjustments for the three potential mediators. The overall percent reduction for maternal depression after adjusting for mediators was 71.4% in PSM analysis. Across all time points, the mediators explained 26.0% (PSM) and 27.2% (IPTW) of the relation between pregnancy intention and parenting stress score (Table 5). The mediators explained 32.1% (PSM) and 33.1% (IPTW) of the relation between pregnancy intention and parenting stress score at 1 year postpartum, and 19.3% (PSW) and 21.5% (IPTW) of the relation between pregnancy intention and parenting stress score at 2 years postpartum. In addition, Additional file 1: Table S5) shows that, across all time points, the mediators explained 81.5% (IPTW) of the relation between pregnancy intention and maternal depression score (Additional file 1: Table S5).

**Table 5:** **Role of examined mediators in the relation between unintended pregnancy and maternal mental health**

	PSM			IPTW		
Depressive symptoms	OR1	OR2	% change	OR1	OR2	% change
1 year postpartum	1.19	1.10	47.4	1.15	1.05	66.7
2 years postpartum	0.95	0.93	NA	1.01	0.95	NA
Overall pregnancy intention	1.07	1.02	71.4	1.08	1.00	100.0
**Parenting stress score**	β1	β2	% change	β1	β2	% change
1 year postpartum	1.09	0.74	32.1	1.24	0.83	33.1
2 years postpartum	0.88	0.71	19.3	1.35	1.06	21.5
Overall pregnancy intention	0.96	0.71	26.0	1.25	0.91	27.2

Note: mediators were marital conflict, fathers’ participation in childcare, and mothers’ knowledge of infant development.

OR1: odds ratio in base model; OR2: odds ratio in model adjusted for mediating variables; β1: mean difference in base model; β2: mean difference in model adjusted for mediating variables.

% change was calculated as (OR1-OR2)/(OR1-1)*100, or (β1-β2)/(β1)*100.

The role of each mediator in the relation between unintended pregnancy and maternal mental health is shown in the Additional file 1: Table S6-8). In general, marital conflict was a stronger mediator than fathers’ participation in childcare and mothers’ knowledge of infant development. The role of knowledge of infant development was minimal.

## Discussion

The results of this study showed that an absence of intention for a pregnancy had an adverse effect on maternal depression and parenting stress, and that the relation between pregnancy intention and maternal mental health was partly mediated by marital conflict, fathers’ participation in child care, and mothers’ knowledge of infant development. These findings were obtained from causal analyses (PSM and IPTW) of longitudinal follow-up data obtained from a national sample of an Asian population. Many prior investigations have reported an association between unintended pregnancy and maternal mental health [4,18,21,23,50], but the associations were often assumed to be non-causal or due to confounding variables [3]. The results of our PSM and IPTW analyses provide support for a causal relation between pregnancy intention and maternal mental health in a non-Western population.

We hypothesized that the magnitude of the relation between pregnancy intention and maternal mental health would decrease with time. The results of this study showed statistically significant differences in the prevalence of maternal depression according to pregnancy intention at the first three time points (from antenatal to 4 months postpartum), but no difference at 1 year or 2 years postpartum. These results support our hypothesis, although there was no significant interaction between pregnancy intention and the time of inquiry on the prevalence of maternal depression or the maternal depression score, this might be due to fluctuating non-linear patterns in the effect of pregnancy intention on maternal depression over the five time points. Several international studies have reported an increased risk of antenatal and postpartum depression for women with unintended pregnancy [4,18,21,23]. Most of these studies examined postpartum depression from a few days to 9 months postpartum, but a few studies have explored the long-term effect of pregnancy intention on postpartum depression. Christensen and colleagues reported that the difference in mean depressive symptom score between low-income Hispanic women with intended and unintended pregnancies was lower at 12 months postpartum than at 4 months postpartum [28], and an Australian study reported that the impact of pregnancy intention on maternal depression diminished over the perinatal period [30]. These studies showed abating trend, which is similar to our results.

In this study, we found that women with an unintended pregnancy reported higher levels of parenting stress over the study period. An Irish cohort study reported that women with unintended pregnancy were more likely to have a high parenting stress level at 9 months postpartum than women with intended pregnancy [4]. A U.S. study explored the parenting stress of mothers when the child was approximately one-year-old according to whether or not they considered aborting the pregnancy, and reported that mothers who considered an abortion had a higher parenting stress score than women who did not consider an abortion [51]. Another U.S. study reported that women who had a later-born child with an unplanned pregnancy tended to experience more parenting stress than women who had a later-born child with a planned pregnancy over the first 3 years after childbirth [50]. These studies showed similar results to our study that the relation between pregnancy intention and parenting stress lasted for a couple of years.

We hypothesized that the magnitude of the relation between pregnancy intention and maternal mental health would decrease with time. One of the challenges that arises from our findings is to explain why the effect of pregnancy intention on maternal depression diminished with time but the differences in parenting stress did not. These differences might be due to the differences in the nature of the two measures. The questions on maternal depression measure the internal emotional status of mothers, whereas the questions on parenting stress are more closely related to external sources of stressors such as economic burden and childcare hassles. The presence of external stressors associated with unintended pregnancy and subsequent childbirth may have a sustained effect on parenting stress, in contrast to internal emotional problems caused by unintended pregnancy, which diminish with time. The results of this study suggest that maternal depression and parenting stress are both important aspects of maternal mental health that are associated with unintended pregnancy and that they may affect childcare and child development in the first year after birth, but beyond the first postpartum year, reducing parenting stress should be the focus for women with unintended pregnancy. Further study is needed to confirm these findings.

We hypothesized that marital conflict, fathers’ participation in child care, and mothers’ knowledge of infant development would mediate the relation between pregnancy intention and maternal mental health. Our results showed that the relation between pregnancy intention and maternal mental health was partially explained by these variables. This supports a previous study that marital relation was a significant predictor of postpartum depression [31]. Our analysis showed that pregnancy intention was strongly associated with marital conflict, and that marital conflict explained more of the relation between pregnancy intention and maternal mental health than either of the other two mediators. These results suggest that unintentional pregnancies may have an adverse effect on later maternal mental health as they brought about marital conflict and lowered the fathers’ participation in childcare, which eventually contributed to perinatal depression and parenting stress in mothers. A mother’s knowledge of infant development affects her confidence of infant care [31,32]. However, our analysis showed that a mother’s knowledge of infant development had a minimal role as mediator of the relation between pregnancy intention and maternal mental health. This is partly because the mother’s knowledge of infant development was not strongly related to pregnancy intention.

Another interesting question from this study would be what additional variables mediate the relation between pregnancy intention and maternal mental health. The three mediators examined in this study explained approximately 26-27% of the relation between unintended pregnancy and parenting stress, and a future challenge is to identify the additional variables that mediate the relation between pregnancy intention and postpartum parenting stress. Candidate variables are problems related to unpreparedness for giving birth, such as monetary preparedness. However, several socioeconomic status variables were considered in the analyses. Further research is required to identify potential mediators.

In this study, results showed that pregnancy intention had an effect on maternal mental health, however, it should be noted that the attributable risk for pregnancy intention on maternal mental health would not be great. For example, overall odds ratios of pregnancy intention on maternal depressive symptoms were 1.20-1.22 and the value of Cohen’s d for parenting stress scores in each year ranged from 0.20 to 0.25. Thus, even though three mediators explained more than one-fourth of the magnitude of the relationship between unintended pregnancy and parenting stress, the absolute reduction of odds and beta by mediators would be small, considering the relatively small impact of pregnancy intention on maternal mental health in the baseline model.

In this study, the prevalence of maternal depression and the maternal depression score were lower in the first month postpartum than in the antenatal period (measured immediately after birth). It is uncertain why maternal depression decreased in the first month postpartum, when postpartum blues and postpartum depression are prevalent. However, it should be noted that the survey related to the first month postpartum was conducted via telephone, but the other surveys were conducted via face-to-face interviews. Mothers might have better disclosed their emotional problems in face-to-face interviews than in telephone surveys. The parenting stress score increased from 4 months to 2 years postpartum, and the increase was due to an increases in the scores for the following questions: “I feel bad because it seems to be my fault when my baby appears emotionally unstable”, “I have difficulty being friendly and warm toward my child”, and “I get irritated if my child pesters me to play with him or her when I am tired”. These three questions accounted for 62.6% of the increase in parenting stress score (data not shown). High scores on these questions indicate a dysfunctional parent–child interaction. An interactive mother-child relation might strengthen the attachment and intimacy, and affect the child’s emotional development.

In this study, we only focused on the mother’s intention. When we conducted sensitivity analysis using different approaches to pregnancy intention (unintended by mother, unintended by mother and father, and unintended by mother or father), the results were generally similar, but the interaction between pregnancy intention and time of inquiry for depressive symptoms was statistically significant in PSM analysis when the pregnancy was unintended by mother or father (see Additional file 1: Table S9). Most prior studies used maternal pregnancy intentions [3], however, further studies are needed to explore consequences of disagreement on pregnancy intention between partners or the role of partner’s intentions on maternal mental health.

This study has limitations. First, the question on pregnancy intention did not distinguish if the pregnancy was unwanted, mistimed, or unplanned, and pregnancy intention was assessed after delivery. Pregnancy intentions can be viewed as a spectrum and thus may be measured with continuous variables to capture doubt about clearly defined intention of pregnancy [52]. In 2005, the estimated induced abortion rate in Korea was 29.8 per 1000 women, and women with unwanted pregnancies were more likely to terminate their pregnancy with induced abortion; therefore, the rate of unintended pregnancy may be underestimated [53,54]. Second, antenatal depression was not assessed during pregnancy but was examined shortly after the birth of a child, when women were asked questions regarding the 30 days prior to giving birth. The antenatal depression status may therefore be inaccurate and may hamper causality for the relationship between unintended pregnancy and antenatal depression. Third, perinatal depression was measured with the K6 and not with structured clinical interview or another more popular tool; therefore, caution should be exercised when comparing our results with other studies. Fourth, women with mental health problems might be more likely to have unintended pregnancy than women without mental health problems. Hall and colleagues showed that, among young women aged 18–20 years with no intention of pregnancy, women with stress or depression and stress at baseline had higher risks of pregnancy over the course of 1 year [55]. A longitudinal study showed that males and females who had depressive symptoms in their adolescent periods were more likely to report an unintended first birth between the ages of 18 and 24 [56]. The insufficient contraception might be related to the risk of unintended pregnancy among depressed women. Women with elevated depression and stress were more likely to be at risk for inconsistent contraceptive use [57]. Although we employed causal analyses in this study, we cannot completely exclude the possibility of this reverse causation because we did not assess pre-conception mental health status.

This study has several strengths. We used a prospective cohort data from a nationally representative sample of a non-Western population. Many of the existing studies on the adverse effect of unintended pregnancy were conducted in a Western population [3]. We used longitudinal data assessed by repeated observations, with consistent measurement of maternal depression and parenting stress for two years after childbirth, and we conducted analysis using PSM and IPTW, which allowed us to reduce confounding effects and estimate causal effects based on the assumption that there is no unmeasured confounders [58,59]. We also provide evidence on the role of mediators in the relation between pregnancy intention and maternal mental health.

## Conclusions

The results suggested that reducing unintended pregnancy as well as increasing fathers’ participation in childcare and decreasing marital conflict might be helpful in improving maternal mental health. Future studies should examine longer term effects of unintended pregnancy on maternal mental health.

## Additional file

Additional file 1: **Survey questionnaires. Table S1.** Baseline covariates after propensity score matching: standardized difference of mean (%) between matched pair for each of the baseline variables. **Table S2.** Crude prevalence of depression and mean depression and parenting stress scores according to pregnancy intention. **Table S3.** Difference in maternal depression score according to pregnancy intention. **Table S4.** The relationships between mediating variables and outcome variables. **Table S5.** Role of examined mediators in the relation between unintended pregnancy and maternal depression score. **Table S6.** Role of marital conflict as a mediator in the relation between pregnancy intention and maternal mental health. Percent reduction in odds ratio of maternal depression and difference in mean maternal depression and parenting stress scores after adjustments for mediators. **Table S7.** Role of fathers’ participation in childcare as a mediator in the relation between pregnancy intention and maternal mental health. Percent reduction in odds ratio of maternal depression and difference in mean maternal depression and parenting stress scores after adjustments for mediators. **Table S8.** Role of mother’s knowledge of infant development as a mediator in the relations between pregnancy intention and maternal mental health. Percent reduction in odds ratio of maternal depression and difference in maternal depression and parenting stress scores after adjustments for mediators. **Table S9.** Results of the sensitivity analysis on the classification of pregnancy intention.

## Abbreviations

PSKC: Panel study on korean children
K6: Kessler 6-item psychological distress scales
PSM: Propensity score matching
IPTW: Inverse probability of treatment weight
