# Supplementary material for: Impact of unintended pregnancy on maternal mental health: a causal analysis using follow up data of the Panel Study on Korean Children (PSKC)
Source: BMC Pregnancy Childbirth. 2015 Apr 3;15:85. doi: 10.1186/s12884-015-0505-4 (PMC4387588; doi:10.1186/s12884-015-0505-4)
Supplement: Additional file 1: — Survey questionnaires. Table S1. Baseline covariates after propensity score matching: standardized difference of mean (%) between matched pair for each of the baseline variables. Table S2. Crude prevalence of depression and mean depression and parenting stress scores according to pregnancy intention. Table S3. Difference in maternal depression score according to pregnancy intention. Table S4. The relationships between mediating variables and outcome variables. Table S5. Role of examined mediators in the relation between unintended pregnancy and maternal depression score. Table S6. Role of marital conflict as a mediator in the relation between pregnancy intention and maternal mental health. Percent reduction in odds ratio of maternal depression and difference in mean maternal depression and parenting stress scores after adjustments for mediators. Table S7. Role of fathers’ participation in childcare as a mediator in the relation between pregnancy intention and maternal mental health. Percent reduction in odds ratio of maternal depression and difference in mean maternal depression and parenting stress scores after adjustments for mediators. Table S8. Role of mother’s knowledge of infant development as a mediator in the relations between pregnancy intention and maternal mental health. Percent reduction in odds ratio of maternal depression and difference in maternal depression and parenting stress scores after adjustments for mediators. Table S9. Results of the sensitivity analysis on the classification of pregnancy intention. [file 12884_2015_505_MOESM1_ESM.docx]

**Additional file 1: Survey questionnaires**

**Depression questionnaire**

Immediately after childbirth: How often have you felt the following feelings during the month right before delivery?

One month postpartum: How often have you felt the following feelings during the month right after childbirth?

Four months, 1 year and 2 years postpartum: How often have you felt the following feelings in the last 30 days?

| How often have you felt … | Never | Rarely | Sometimes | Often | Always |
| --- | --- | --- | --- | --- | --- |
| 1) Anxious? | ① | ② | ③ | ④ | ⑤ |
| 2) Lethargic? | ① | ② | ③ | ④ | ⑤ |
| 3) Impatient? | ① | ② | ③ | ④ | ⑤ |
| 4) Difficulty doing anything? | ① | ② | ③ | ④ | ⑤ |
| 5) Persistently sad even if you try many things to cheer up? | ① | ② | ③ | ④ | ⑤ |
| 6) That you are worthless? | ① | ② | ③ | ④ | ⑤ |

**Parenting stress questionnaire**

These are the difficulties mothers experience in caring for their baby. Please mark the applicable item for each based on your experiences.

| Item | Never | Rarely | Sometimes | Often | Always |
| --- | --- | --- | --- | --- | --- |
| 1) I am not sure whether I will become a good parent. | ① | ② | ③ | ④ | ⑤ |
| 2) I am not sure whether I can raise my child well. | ① | ② | ③ | ④ | ⑤ |
| 3) I feel my child lags behind others because I am not properly performing my role as a parent. | ① | ② | ③ | ④ | ⑤ |
| 4) I want to escape my child. | ① | ② | ③ | ④ | ⑤ |
| 5) I have difficulty being friendly and warm toward my child. | ① | ② | ③ | ④ | ⑤ |
| 6) I feel confused because there is a lot of child care-related information to choose from. | ① | ② | ③ | ④ | ⑤ |
| 7) My life is not as enjoyable after having my child. | ① | ② | ③ | ④ | ⑤ |
| 8) I feel burdened by childcare costs. | ① | ② | ③ | ④ | ⑤ |
| 9) I feel bad because it seems to be my fault when my baby appears emotionally unstable. | ① | ② | ③ | ④ | ⑤ |
| 10) I get irritated if my child pesters me to play with him or her when I am tired. | ① | ② | ③ | ④ | ⑤ |

Note: At the 2 year postpartum time point, one item was added to this questionnaire (“When I go to a party I usually expect not to enjoy myself”) but was excluded our analysis.

**Marital conflict questionnaire**

These are things that happen to married couples. Please mark the most applicable item for each based on what you and your husband experience.

| Item | Never | Rarely | Sometimes | Often | Always |
| --- | --- | --- | --- | --- | --- |
| 1) Little arguments escalate into ugly fights with accusations, criticisms, name-calling, or bringing up past hurts. | ① | ② | ③ | ④ | ⑤ |
| 2) My husband criticizes or belittles my opinions, feelings, or desires. | ① | ② | ③ | ④ | ⑤ |
| 3) My husband seems to view my words or actions more negatively than I mean them to be. | ① | ② | ③ | ④ | ⑤ |
| 4) My husband is unlikely to respect me. | ① | ② | ③ | ④ | ⑤ |
| 5) It is no use telling my husband what I really think, feel, or desire to change in our relationship. | ① | ② | ③ | ④ | ⑤ |
| 6) I seriously think about how things would have turned out had I married another man. | ① | ② | ③ | ④ | ⑤ |
| 7) I feel lonely in this relationship. | ① | ② | ③ | ④ | ⑤ |
| 8) When we argue, one of us withdraws – that is, doesn’t want to talk about it anymore or leaves the scene. | ① | ② | ③ | ④ | ⑤ |

**Fathers’ participation in childcare questionnaire**

These questions deal with the cooperation of your husband in childcare. Please mark the most applicable item for each.

| Item | Never | Rarely | Sometimes | Often | Always |
| --- | --- | --- | --- | --- | --- |
| 1) My husband buys toys and things for our baby. | ① | ② | ③ | ④ | ⑤ |
| 2) My husband takes an interest in the habits or behaviors of our baby and instructs him or her. | ① | ② | ③ | ④ | ⑤ |
| 3) My husband feeds or bathes our baby. | ① | ② | ③ | ④ | ⑤ |
| 4) My husband plays with our baby. | ① | ② | ③ | ④ | ⑤ |

**Knowledge of infant development questionnaire**

These are statements on the normal development and care of babies. Please mark the most applicable item for each based on your general knowledge of babies instead of what you know about your child.

| Item | Yes | No | Not sure |
| --- | --- | --- | --- |
| 1) Even after correcting a child on a word, he or she often keeps using the wrong word for a while. |  |  |  |
| 2) A child understands only the words he or she can use. |  |  |  |
| 3) If a child becomes shy or fretful, it usually means that he or she has an emotional issue. |  |  |  |
| 4) The method of caring for a child rarely impacts his or her intelligence. |  |  |  |
| 5) If a child is very young, he or she rarely affects his or her parents or their parenting style. |  |  |  |
| 6) If the child becomes an older sibling, he or she sometimes sucks a finger or urinates while sleeping. |  |  |  |
| 7) Premature babies are mostly abused or neglected, or they end up being mentally retarded. ("Premature babies" mean babies who were born weighting 2.5 kg or less and before 37 weeks of pregnancy.) |  |  |  |
| 8) Even healthy babies sometimes spit out food if it’s new to them until they get used to it. |  |  |  |
| 9) The personality or disposition of a baby is decided within 6 months of birth; after that, babies hardly change. |  |  |  |
| 10) The reaction of parents to their baby within a few months of birth determines whether the baby grows up to be a happy, positive child or a depressed, negative one. |  |  |  |
| 11) A child obtains all of his or her words by mimicking the older people surrounding him or her. |  |  |  |
| 12) If it’s too noisy, or there are many things to look at, a baby cannot focus on the things around him or her. |  |  |  |
| 13) Even some normal babies do not like being cradled. |  |  |  |

**Table S1**. Baseline covariates after propensity score matching: standardized difference of mean (%) between matched pair for each of the baseline variables.

| PS matching | Total | Pregnancy intention | | Standardized Difference of Mean (%) |
| --- | --- | --- | --- | --- |
|  |  | Unintended | Intended |  |
|  | N = 1042 | N = 521 | N = 521 |  |
| Maternal age (years) | 31.0 ± 3.9 | 31.0 ± 4.2 | 31.0 ± 3.6 | 1.20% |
| Paternal age (years) | 33.7 ± 4.2 | 33.8 ± 4.6 | 33.6 ± 3.7 | 3.50% |
| Infant’s sex |  |  |  |  |
| Boy | 533 (51.2) | 260 (49.9) | 273 (52.4) | 4.99% |
| Girl | 509 (48.9) | 261 (50.1) | 248 (47.6) |  |
| Infant’s birth order |  |  |  |  |
| First born | 399 (38.3) | 200 (38.4) | 199 (38.2) | 0.40% |
| Later born | 643 (61.7) | 321 (61.6) | 322 (61.8) |  |
| Maternal education |  |  |  |  |
| University or over | 381 (36.6) | 196 (37.6) | 185 (35.5) | 4.38% |
| Junior college | 294 (28.2) | 145 (27.8) | 149 (28.6) | 1.71% |
| High school or under | 367 (35.2) | 183 (34.6) | 187 (35.9) | 1.60% |
| Paternal education |  |  |  |  |
| University or over | 492 (47.2) | 246 (47.2) | 246 (47.2) | 0.00% |
| Junior college | 227 (21.8) | 114 (21.9) | 113 (21.7) | 0.46% |
| High school or under | 323 (31.0) | 161 (30.9) | 162 (31.1) | 0.42% |
| Maternal occupation |  |  |  |  |
| Non-manual | 269 (25.8) | 130 (30.0) | 139 (26.7) | 3.95% |
| Manual | 67 (6.4) | 33 (6.3) | 34 (6.5) | 0.78% |
| Housewife/others | 706 (67.8) | 358 (68.7) | 348 (66.8) | 4.11% |
| Paternal occupation |  |  |  |  |
| Non-manual | 644 (61.8) | 322 (61.8) | 322 (61.8) | 0.00% |
| Manual | 398 (38.2) | 199 (38.2) | 199 (38.2) |  |
| Household income quintiles |  |  |  |  |
| I (highest) | 176 (16.9) | 89 (17.1) | 87 (16.7) | 1.02% |
| II | 193 (18.5) | 95 (18.2) | 98 (18.8) | 1.48% |
| III | 195 (18.7) | 100 (19.2) | 95 (18.2) | 2.46% |
| IV | 215 (20.6) | 105 (20.2) | 110 (21.1) | 2.37% |
| V (lowest) | 263 (25.2) | 132 (25.3) | 131 (25.1) | 0.44% |
| Maternal smoking |  |  |  |  |
| No | 1033 (99.1) | 516 (99.0) | 517 (99.2) | 2.07% |
| Yes | 9 (0.9) | 5 (1.0) | 4 (0.8) |  |
| Paternal smoking |  |  |  |  |
| No | 1174 (56.6) | 263 (50.5) | 271 (52.0) | 3.07% |
| Yes | 902 (43.5) | 258 (49.5) | 250 (48.0) |  |
| Maternal alcohol consumption |  |  |  |  |
| No | 764 (73.3) | 378 (72.6) | 386 (74.1) | 3.47% |
| Yes | 278 (26.7) | 143 (72.6) | 135 (25.9) |  |
| Paternal alcohol consumption |  |  |  |  |
| No | 367 (21.4) | 120 (23.0) | 118 (22.7) | 0.91% |
| Yes | 1350 (78.6) | 401 (77.0) | 403 (77.3) |  |
| Propensity Score | -1.00 ± 0.40 | -1.00 ± 0.40 | -1.00 ± 0.40 | 0.10% |

**Table S2.** Crude prevalence of depression and mean depression and parenting stress scores according to pregnancy intention.

|  | Depressive symptoms† | | Depression score | | Parenting stress score | |
| --- | --- | --- | --- | --- | --- | --- |
|  | Intended pregnancy | Unintended pregnancy | Intended pregnancy | Unintended pregnancy | Intended pregnancy | Unintended pregnancy |
| Antenatal‡ | 381 (24.77) | 156 (29.89) | 11.24 ±3.77 | 11.75 ± 4.33 |  |  |
| 1 month postpartum | 126 (10.48) | 60 (14.35) | 8.99 ±3.54 | 9.36 ± 3.76 |  |  |
| 4 months postpartum | 368 (26.80) | 160 (33.68) | 11.50 ±4.08 | 12.24 ± 4.57 | 27.07 ± 6.05 | 28.46 ± 6.48 |
| 1 year postpartum | 360 (26.24) | 147 (31.61) | 11.38 ±4.09 | 11.83 ± 4.54 | 26.87 ± 6.33 | 28.42 ± 6.30 |
| 2 years postpartum | 380 (29.76) | 131 (30.54) | 11.73 ±4.14 | 12.02 ± 4.26 | 27.78 ± 6.44 | 29.05 ± 6.18 |

†Depression score ≥14.

‡ Antenatal depression was measured immediately after childbirth

Data are N (%) or mean ± standard deviation.

**Table S3.** Difference in maternal depression score according to pregnancy intention.

|  | PSM | | | IPTW | | |
| --- | --- | --- | --- | --- | --- | --- |
|  | β (SE) | 95% CI | P value | β (SE) | 95% CI | P value |
| **Depression score** |  |  |  |  |  |  |
| Time of inquiry |  |  |  |  |  |  |
| Antenatal* | 0.50 (0.25) | 0.02–0.99 | 0.0424 | 0.33 (0.22) | -0.10–0.76 | 0.1296 |
| 1 month postpartum | 0.24 (0.25) | -0.25–0.73 | 0.3354 | 0.50 (0.22) | 0.07–0.93 | 0.0225 |
| 4 months postpartum | 0.52 (0.27) | -0.01–1.04 | 0.0527 | 0.67 (0.24) | 0.20–1.14 | 0.0052 |
| 1 year postpartum | 0.13 (0.27) | -0.40–0.66 | 0.6327 | 0.17 (0.23) | -0.29–0.62 | 0.4765 |
| 2 years postpartum | 0.02 (0.30) | -0.56–0.60 | 0.9426 | 0.29 (0.25) | -0.20–0.79 | 0.2407 |
| Pregnancy intention*time of inquiry^§^ |  |  | 0.5036 |  |  | 0.3054 |
| Overall pregnancy intention | 0.28 (0.11) | 0.06–0.49 | 0.0133 | 0.39 (0.16) | 0.08–0.70 | 0.0135 |

*Antenatal depression was measured immediately after childbirth. ^§^The interaction between pregnancy intention and time of inquiry.

PSM: propensity score matching; IPTW: inverse probability of treatment weighted; CI: confidence interval.

**Table S4**. The relationships between mediating variables and outcome variables.

|  | Marital conflict | | Fathers’ participation in child care | | Knowledge of infant development | |
| --- | --- | --- | --- | --- | --- | --- |
|  | β (SE) | P value | β (SE) | P value | β (SE) | P value |
| Maternal depressive symptoms |  |  |  |  |  |  |
| 1 year postpartum | 0.02 (0.00) | <.0001 | -0.02 (0.00) | <.0001 | -0.02 (0.01) | 0.0022 |
| 2 years postpartum | 0.02 (0.00) | <.0001 | -0.01 (0.00) | 0.0002 | -0.02 (0.01) | 0.0007 |
| Maternal depression score |  |  |  |  |  |  |
| 1 year postpartum | 0.25 (0.02) | <.0001 | -0.19 (0.03) | <.0001 | -0.18 (0.06) | 0.001 |
| 2 years postpartum | 0.24 (0.02) | <.0001 | -0.17 (0.04) | <.0001 | -0.19 (0.06) | 0.0014 |
| Parenting stress scores |  |  |  |  |  |  |
| 1 year postpartum | 0.32 (0.03) | <.0001 | -0.25 (0.05) | <.0001 | -0.35 (0.08) | <.0001 |
| 2 years postpartum | 0.30 (0.03) | <.0001 | -0.26 (0.05) | <.0001 | -0.34 (0.09) | 0.0001 |

**Table S5**. Role of examined mediators in the relation between unintended pregnancy and maternal depression score.

|  | PSM | | | IPTW | | |
| --- | --- | --- | --- | --- | --- | --- |
| **Depression score** | β1 | β2 | % change | β1 | β2 | % change |
| 1 year postpartum | 0.13 | -0.08 | NA | 0.17 | -0.08 | NA |
| 2 years postpartum | 0.02 | -0.15 | NA | 0.29 | 0.13 | 55.2 |
| Overall pregnancy intention | 0.09 | -0.09 | NA | 0.27 | 0.05 | 81.5 |

Note: mediators were marital conflict, fathers’ participation in childcare, and mothers’ knowledge of infant development.

β1: mean difference in base model; β2: mean difference in model adjusted for mediating variables. % change was calculated as (β1-β2)/(β1)*100.

**Table S6.** Role of marital conflict as a mediator in the relation between pregnancy intention and maternal mental health. Percent reduction in odds ratio of maternal depression and difference in mean maternal depression and parenting stress scores after adjustments for mediators.

|  | PSM | | | IPTW | | |
| --- | --- | --- | --- | --- | --- | --- |
| Depression | OR1 | OR2 | % change | OR1 | OR2 | % change |
| 1 year postpartum | 1.19 | 1.11 | 40.2 | 1.15 | 1.06 | 62.9 |
| 2 years postpartum | 0.95 | 0.94 | NA | 1.01 | 0.96 | NA |
| Overall | 1.07 | 1.03 | 57.1 | 1.17 | 1.01 | 94.1 |
|  |  |  |  |  |  |  |
| Depression score | β1 | β2 | % change | β1 | β2 | % change |
| 1 year postpartum | 0.13 | -0.07 | NA | 0.16 | -0.08 | NA |
| 2 years postpartum | 0.02 | -0.09 | NA | 0.29 | 0.19 | 36.2 |
| Overall | 0.09 | -0.05 | NA | 0.41 | 0.08 | 80.5 |
|  |  |  |  |  |  |  |
| Parenting stress score | β1 | β2 | % change | β1 | β2 | % change |
| 1 year postpartum | 1.09 | 0.74 | 31.4 | 1.24 | 0.83 | 32.7 |
| 2 years postpartum | 0.88 | 0.71 | 19.1 | 1.35 | 1.06 | 21.4 |
| Overall | 0.96 | 0.79 | 17.7 | 1.16 | 0.97 | 16.4 |

PSM: propensity score matching. IPTW: inverse probability of treatment weighted.

OR1: odds ratio in base model; OR2: odds ratio in model adjusted for mediating variables; β1: mean difference in base model; β2: mean difference in model adjusted for mediating variables.

% change was calculated as (OR1-OR2)/(OR1-1)*100, or (β1-β2)/(β1)*100.

**Table S7**. Role of fathers’ participation in childcare as a mediator in the relation between pregnancy intention and maternal mental health. Percent reduction in odds ratio of maternal depression and difference in mean maternal depression and parenting stress scores after adjustments for mediators.

|  | PSM | | | IPTW | | |
| --- | --- | --- | --- | --- | --- | --- |
| Depression | OR1 | OR2 | % change | OR1 | OR2 | % change |
| 1 year postpartum | 1.19 | 1.11 | 38.7 | 1.15 | 1.06 | 60.6 |
| 2 years postpartum | 0.95 | 0.97 | 42.3 | 1.01 | 0.99 | NA |
| Overall | 1.07 | 1.04 | 42.9 | 1.17 | 1.03 | 82.4 |
|  |  |  |  |  |  |  |
| Depression score | β1 | β2 | % change | β1 | β2 | % change |
| 1 year postpartum | 0.13 | -0.02 | NA | 0.16 | -0.01 | NA |
| 2 years postpartum | 0.02 | -0.01 | NA | 0.29 | 0.26 | 11.7 |
| Overall | 0.09 | 0.01 | 88.9 | 0.41 | 0.15 | 63.4 |
|  |  |  |  |  |  |  |
| Parenting stress score | β1 | β2 | % change | β1 | β2 | % change |
| 1 year postpartum | 1.09 | 0.84 | 22.2 | 1.24 | 0.93 | 24.9 |
| 2 years postpartum | 0.88 | 0.80 | 8.5 | 1.35 | 1.19 | 11.4 |
| Overall | 0.96 | 0.81 | 15.6 | 1.16 | 1.00 | 13.8 |

PSM: propensity score matching. IPTW: inverse probability of treatment weighted.

OR1: odds ratio in base model; OR2: odds ratio in model adjusted for mediating variables; β1: mean difference in base model; β2: mean difference in model adjusted for mediating variables.

% change was calculated as (OR1-OR2)/(OR1-1)*100, or (β1-β2)/(β1)*100.

**Table S8**. Role of mother’s knowledge of infant development as a mediator in the relations between pregnancy intention and maternal mental health. Percent reduction in odds ratio of maternal depression and difference in maternal depression and parenting stress scores after adjustments for mediators.

|  |  | PSM | | |  | IPTW | | |
| --- | --- | --- | --- | --- | --- | --- | --- | --- |
| Depression |  | OR1 | OR2 | % change |  | OR1 | OR2 | % change |
| 1 year postpartum |  | 1.19 | 1.17 | 7.3% |  | 1.15 | 1.14 | 10.6% |
| 2 years postpartum |  | 0.95 | 1.02 | NA |  | 1.01 | 1.03 | NA |
| Overall |  | 1.07 | 1.10 | NA |  | 1.17 | 1.09 | 47.1 |
|  |  |  |  |  |  |  |  |  |
| Depression score |  | β1 | β2 | % change |  | β1 | β2 | % change |
| 1 year postpartum |  | 0.13 | 0.06 | 54.2% |  | 0.16 | 0.11 | 35.4% |
| 2 years postpartum |  | 0.02 | 0.02 | NA |  | 0.29 | 0.32 | NA |
| Overall |  | 0.09 | 0.07 | 22.2 |  | 0.41 | 0.24 | 41.5 |
|  |  |  |  |  |  |  |  |  |
| Parenting stress score |  | β1 | β2 | % change |  | β1 | β2 | % change |
| 1 year postpartum |  | 1.09 | 1.02 | 6.1% |  | 1.24 | 1.14 | 8.0% |
| 2 years postpartum |  | 0.88 | 0.93 | NA |  | 1.35 | 1.31 | 2.5% |
| Overall |  | 0.96 | 0.96 | 0.00 |  | 1.16 | 1.18 | NA |

PSM: propensity score matching. IPTW: inverse probability of treatment weighted.

OR1: odds ratio in base model; OR2: odds ratio in model adjusted for mediating variables; β1: mean difference in base model; β2: mean difference in model adjusted for mediating variables.

% change was calculated as (OR1-OR2)/(OR1-1)*100, or (β1-β2)/(β1)*100.

**Table S9**. Results of the sensitivity analysis on the classification of pregnancy intention.

|  | PSM | | | IPTW | | |
| --- | --- | --- | --- | --- | --- | --- |
|  | OR | 95% CI | P value | OR | 95% CI | P value |
| ***Depressive symptoms*** |  |  |  |  |  |  |
| **Unintended by mother (521 pair)** |  |  |  |  |  |  |
| Pregnancy intention*time of inquiry |  |  | 0.1819 |  |  | 0.2402 |
| Overall pregnancy intention | 1.22 | 1.02–1.46 | 0.0290 | 1.20 | 1.03–1.40 | 0.0212 |
| **Unintended by mother and father (369 pair)** |  |  |  |  |  |  |
| Pregnancy intention*time of inquiry |  |  | 0.6586 |  |  | 0.5306 |
| Overall pregnancy intention | 1.13 | 0.92-1.40 | 0.2499 | 1.12 | 0.93-1.34 | 0.2282 |
| **Unintended by mother or father (569 pair)** |  |  |  |  |  |  |
| Antenatal | 1.24 | 0.96-1.61 | 0.1030 | - | - | - |
| 1 month postpartum | 2.26 | 1.43-3.55 | 0.0004 | - | - | - |
| 4 months postpartum | 1.25 | 0.96-1.62 | 0.1009 | - | - | - |
| 1 year postpartum | 0.99 | 0.75-1.29 | 0.9281 | - | - | - |
| 2 years postpartum | 1.09 | 0.81-1.45 | 0.5663 | - | - | - |
| Pregnancy intention*time of inquiry |  |  | 0.0205 |  |  | 0.3298 |
| Overall pregnancy intention | - | - | - | 1.20 | 1.03-1.39 | 0.018 |
|  |  |  |  |  |  |  |
|  | β (SE) | 95% CI | P value | β (SE) | 95% CI | P value |
| ***Parenting stress score*** |  |  |  |  |  |  |
| **Unintended by mother (521 pair)** | 0.85 (0.21) | 0.44–1.27 | <0.0001 | 1.16 (0.30) | 0.58–1.74 | <0.0001 |
| **Unintended by mother and father (369 pair)** | 1.03 (0.25) | 0.54-1.52 | <0.0001 | 1.34 (0.32) | 0.71-1.97 | <0.0001 |
| **Unintended by mother or father (569 pair)** | 0.64 (0.21) | 0.23-1.05 | 0.0020 | 0.93 (0.28) | 0.38-1.48 | 0.0009 |
